# Cardiorespiratory Polygraphy for Detection of Obstructive Sleep Apnea in Patients With Atrial Fibrillation

**DOI:** 10.3389/fcvm.2021.758548

**Published:** 2021-11-30

**Authors:** Michiel Delesie, Lieselotte Knaepen, Johan Verbraecken, Karolien Weytjens, Paul Dendale, Hein Heidbuchel, Lien Desteghe

**Affiliations:** ^1^Research Group Cardiovascular Diseases, University of Antwerp, Antwerp, Belgium; ^2^Antwerp University Hospital, Edegem, Belgium; ^3^Faculty of Medicine and Life Sciences, Hasselt University, Hasselt, Belgium; ^4^Heart Centre Hasselt, Jessa Hospital, Hasselt, Belgium; ^5^Department of Pulmonary Medicine and Multidisciplinary Sleep Disorders Centre, Antwerp University Hospital and Research Group Laboratory of Experimental Medicine and Pediatrics, Faculty of Medicine and Health Sciences, University of Antwerp, Edegem, Belgium; ^6^Sleep Centre Hasselt, Jessa Hospital, Hasselt, Belgium

**Keywords:** atrial fibrillation, sleep apnea, polygraphy, screening, apnea–hypopnea index (AHI)

## Abstract

**Background:** Obstructive sleep apnea (OSA) is a modifiable risk factor of atrial fibrillation (AF) but is underdiagnosed in these patients due to absence of good OSA screening pathways. Polysomnography (PSG) is the gold standard for diagnosing OSA but too resource-intensive as a screening tool. We explored whether cardiorespiratory polygraphy (PG) devices using an automated algorithm for Apnea-Hypopnea Index (AHI) determination can meet the requirements of a good screening tool in AF patients.

**Methods:** This prospective study validated the performance of three PGs [ApneaLink Air (ALA), SOMNOtouch RESP (STR) and SpiderSAS (SpS)] in consecutive AF patients who were referred for PSG evaluation. Patients wore one of the three PGs simultaneously with PSG, and a different PG during each of three consecutive nights at home. Severity of OSA was classified according to the AHI during PSG (<5 = no OSA, 5–14 = mild, 15–30 = moderate, >30 = severe).

**Results:** Of the 100 included AF patients, PSG diagnosed at least moderate in 69% and severe OSA in 33%. Successful PG execution at home was obtained in 79.1, 80.2 and 86.8% of patients with the ALA, STR and SpS, respectively. For the detection of clinically relevant OSA (AHI ≥ 15), an area under the curve of 0.802, 0.772 and 0.803 was calculated for the ALA, STR and SpS, respectively.

**Conclusions:** This study indicates that home-worn PGs with an automated AHI algorithm can be used as OSA screening tools in AF patients. Based on an appropriate AHI cut-off value for each PG, the device can guide referral for definite PSG diagnosis.

## Introduction

Obstructive sleep apnea (OSA) influences the progression of atrial fibrillation (AF) with a 1.9-fold increased risk of AF recurrence in AF patients with OSA compared to those without (1). Moreover, OSA treatment with Continuous Positive Airway Pressure (CPAP) can influence AF evolution by increasing the response to AF antiarrhythmic drugs and better maintenance of sinus rhythm after rhythm restoring procedures (2–4).

The prevalence of OSA in AF patients is variable, depending on diagnosing technique and type of AF, with estimates between 21 and 85%. (4–6) In clinical practise, however, OSA is a highly underrecognised and undertreated as AF risk factor, partly due to the lack of easily accessible screening tools and strategies.

According to the 2020 European Society of Cardiology (ESC) guidelines for the diagnosis and management of AF, testing for OSA is reasonable before initiation of rhythm control therapy in symptomatic AF patients (7). However, the authors do not propose any screening strategy for OSA in this patient population. OSA screening questionnaires and scales are sometimes used in the general population or specific patient populations (e.g., surgical patients), but performance is insufficient to screen for OSA in AF patients. (8–10) Currently, polysomnography (PSG) is the accepted gold standard for diagnosing OSA. Additionally, OSA diagnosis by PSG is mandatory before starting CPAP therapy in several countries (11). However, due to its cost, labour-intensiveness and limited availability, PSG is not the ideal screening method for the large population of AF patients. While in other countries, cardiorespiratory polygraphy (PG) devices (level 3 sleep testing) are also used for diagnosing OSA, it is still recommended that these recordings need manual revision and scoring by trained personnel or sleep physicians (i.e., time-intensive) (12). A great advantage of PGs is that these compact devices can be applied by the patient at home. Moreover, most PG manufacturers provide an automated algorithm software program so that the detected disordered breathing events are readily classified. Combined with their acceptable cost, PGs with automated analysis could meet the requirements of a good OSA detection tool.

Therefore, this prospective study wanted to validate and examine the performance of three different PGs devices combined with automated analysis, as detection tools for OSA in AF patients, compared with the gold standard PSG.

## Methods

The CarpOSAF study (Belgian Registration number: B300201835708) was a prospective, multicentre validation study in which various tools such as questionnaires, scoring systems and different PGs were evaluated as screening options for OSA in AF patients. Patients were included at two Belgian tertiary care centres, the Antwerp University Hospital and the Jessa Hospital Hasselt. The research protocol was approved by the Ethics Committees of the participating centres and the study was conducted in compliance with the Declaration of Helsinki. All patients provided written informed consent.

### Study Population

From May 2018 until November 2020, patients referred for PSG at the two sleep clinics were evaluated for participation in the study. Inclusion criteria were (1) planned PSG for diagnosis of sleep-disordered breathing, (2) history of AF or atrial flutter, with proven diagnosis on an electrocardiogram, and (3) capable of signing the informed consent. Exclusion criteria consisted of (1) not able to speak and read Dutch, (2) age <18 years, (3) physical/cognitive impairment (e.g., severe dementia), and (4) participation in other studies.

### Procedure

For all study patients, a standard workup at the sleep clinic, including clinical measurements such as weight, height, neck- and waist circumference, was conducted. Demographic variables and AF specific characteristics (type of AF, AF duration, CHA_2_DS_2_-VASc, HAS-BLED) were derived from the patients' medical file.

The total duration of the study comprised of four nights (one in hospital, three at home), during which three different (cardio)respiratory PG devices were tested for validation, namely the ApneaLink Air (ALA), the SOMNOtouch RESP (STR) and the SpiderSAS (SpS).

Each patient tested one of the three PG randomly and simultaneously with their planned overnight PSG evaluation. The study personnel conducted proper attachment of the PG to avoid interference with the PSG channels. The following morning, a Comfort Questionnaire (CQ) was filled out by the patient to evaluate the PSG examination (Supplementary Annex 1). On returning home, the study patients took the three PGs so that with the instructions provided in the hospital, they could attach the PGs themselves at home during three consecutive nights. The patients also received detailed manuals with step by step photos about when and how to use the different PG devices. The SpS could only be used during the first night at home due to its limitations in time registration after programmation. The order of the other PG use at home was randomised. After every PG night at home, the patient had to register their time of going to sleep and waking up. They also filled out the CQ regarding the convenience of the tested PG. After completing the three nights with the PGs, the patient returned the equipment back to the centre, where the recordings were analysed by each PG's specific automated algorithm software for detection of sleep-disordered breathing events.

### Polysomnography

As mentioned, all subjects underwent a PSG examination (Natus Schwarzer; Micromed Morpheus at the University Hospital of Antwerp and Medatec Dream at the Jessa Hospital). Data was manually scored by the staff of the sleep clinics according to the American Academy of Sleep Medicine (AASM) 2012 criteria in which an apnea was defined as a decrease in airflow by 90% from baseline for at least 10 s and a hypopnea was defined as a decrease in airflow ≥ 30% from baseline for at least 10 s associated with a decrease of oxygen saturation of at least 3% or an arousal (13). The apnea-hypopnea index (AHI) was the ratio of the number of apneas/hypopneas divided by the hours of the evaluation period. Study patients having an AHI ≥5 events/h were considered having OSA. Severity of OSA was classified according to AHI (<5 = no OSA, 5–14 = mild, 15–30 = moderate, >30 = severe) (13). An AHI ≥ 15 events/h was considered to be clinically relevant OSA, as for this threshold CPAP treatment is indicated and reimbursed in many countries (12).

### Cardiorespiratory Polygraphy Devices and Data Processing

Specifications of each PG and the manufacturers' software are described in Supplementary Table 1.

After transferring the patients' PG recordings to the designated software, a manual review of the channels' signal quality was performed by one study investigator (M.D.) focusing on the parameters nasal flow, respiratory effort and oxygen saturation, as these are contributing to the AHI determination. If one of the three channels failed to record a good signal for a minimum of 4 h, the data were excluded for analysis. This limit was chosen based on the suggestion of the clinical practise guideline for diagnostic testing of OSA, made by the AASM (11). Additionally, for the STR, the ‘wake/sleep' algorithm (based on activity and position analysis) of the DOMINO light software was also used for determining a proper sleep time. For the SpS, the documented time of sleep and getting up of the patients were imported in the SpS recordings as the SYNESCOPE software did not distinguish sleep vs. awake status. As the ALA was manually (de)activated by the study patient and the automated algorithm already excluded the first 10 min and the last 2 min of the recording, no additional adaptations were applied to the recording.

### Statistics

Data were analysed using IBM SPSS version 27.0. Variables were described as numbers and percentages or as mean ± standard deviation, as appropriate. Normal distribution was assessed using the Shapiro-Wilk test. For continuous variables, differences between two groups were compared using the independent *T*-test/paired *T*-test (parametric) and the Mann-Whitney U test/Wilcoxon signed rank test (non-parametric). The chi-squared test was used for categorical variables. Differences between the scores on the different CQ components were analysed using the Friedman test (non-parametric). *P*-values < 0.05 were considered statistically significant.

Sensitivity, specificity, positive predictive value (PPV) and negative predictive value (NPV) were calculated for the different cut-off values predicting risk for OSA of the three PGs at various levels of severity of OSA. The Cohen's Kappa was also calculated between the PGs and the PSG result (for simultaneous and separate measurements) to evaluate the agreement between these measurements for the different AHI cohorts. Receiver operating characteristics (ROC)-curves and corresponding areas under the curve (AUC) were generated for the three PGs to assess their predictive value for OSA in AF patients. These graphs were generated for the PSG groups with AHI thresholds ≥5, ≥15 and >30 events/h. Additionally, a ROC-curve/AUC was calculated for predicting at least moderate OSA, including only non-permanent AF patients. Appropriate AHI cut-off values for each PG were derived from the coordinates of the ROC-curves and determined based on the best discriminating ability to rule in (=high specificity) or rule out (=high sensitivity) clinically relevant OSA (i.e., AHI ≥ 15 events/h), in combination with an optimal Youden's J Index (i.e., sensitivity+specificity-1).

As this was part of a validation study, no specific sample size was calculated. The aim was to include 50 consecutive AF patients, referred for a diagnostic PSG, in each centre, i.e., for a total of 100 patients.

## Results

### Patient Characteristics

A total of 149 sleep clinic patients with a history of AF were evaluated for inclusion in the study. Nineteen patients were excluded, and 30 AF patients refused to participate, or did not show up or their PSG was cancelled (Supplementary Figure 1). A total of 100 AF patients were eventually included in this study.

The mean age of these 100 patients was 64.0 ± 8.7 years and BMI was 30.6 ± 5.9 kg/m^2^ (Table 1). Rhythm control management was pursued in 55.0% and the modified European Heart Rhythm Association AF symptom score (mEHRA) was at least 2A in 64.0%. After PSG evaluation, 90% of the included AF patients had an AHI ≥5 events/h with 21.0, 36.0 and 33.0% having mild, moderate or severe OSA respectively. Hence, 69% had “clinically relevant” OSA.

**Table 1:** Baseline characteristics of the study population.

	**Total study population (*n* = 100)**	**UZ Antwerp (*n* = 49)**	**Jessa Hospital Hasselt (*n* = 51)**	***P*-value between centres**
Age (years), mean ± SD	64.0 ± 8.7	63.8 ± 10.1	64.2 ± 7.2	0.828
Male, n (%)	73 (73.0)	42 (85.7)	31 (60.8)	**0.005**
BMI (kg/m^2^), mean ± SD	30.6 ± 5.9	29.5 ± 5.0	31.6 ± 6.6	0.085
Waist circumference (cm), mean ± SD	108.7 ± 14.2	107.9 ± 14.0	109.5 ± 14.4	0.747
Neck circumference (cm), mean ± SD	41.3 ± 4.0	41.5 ± 3.4	41.2 ± 4.4	0.721
Kind of AF, n (%)				0.212
First diagnosed	14 (14.0)	6 (12.2)	8 (15.7)	
Paroxysmal AF	53 (53.0)	22 (44.9)	31 (60.8)	
Persistent AF	20 (20.0)	12 (24.5)	8 (15.7)	
Permanent AF	13 (13.0)	9 (18.4)	4 (7.8)	
Time since AF diagnosis (years), mean ± SD	5.4 ± 5.6	5.4 ± 5.8	5.4 ± 5.4	0.895
CHA_2_DS_2_-VASc score, mean ± SD	2.4 ± 1.7	2.5 ± 1.9	2.4 ± 1.5	0.831
HAS-BLED score, mean ± SD	1.2 ± 0.9	1.3 ± 0.9	1.2 ± 0.9	0.418
mEHRA ≥ 2a, n (%)	64 (64.0)	27 (55.1)	37 (72.5)	0.069
Referred by cardiologist, n (%)	42 (42.0)	26 (53.1)	16 (33.3)	**0.050**
Anticoagulation therapy, n (%)				0.790
NOAC	58 (58.0)	29 (59.2)	29 (56.9)	
VKA	6 (6.0)	3 (6.1)	3 (5.9)	
None	36 (36.0)	17 (34.7)	19 (37.3)	
Rhythm control, n (%)	55 (55.0)	35 (71.4)	20 (39.2)	**0.001**
OSA Severity				0.964
No OSA (AHI <5), n (%)	10 (10.0)	5 (10.2)	5 (9.8)	
Mild OSA (AHI 5-14), n (%)	21 (21.0)	11 (22.4)	10 (19.6)	
Moderate OSA (AHI 15–30), n (%)	36 (36.0)	18 (36.7)	18 (35.3)	
Severe OSA (AHI>30), n (%)	33 (33.0)	15 (30.6)	18 (35.3)	

*AF, atrial fibrillation; BMI, body mass index; NOAC, non-vitamin K antagonist oral anticoagulant; OSA, obstructive sleep apnea; VKA, vitamin K antagonist; AHI, apnea-hypopnea index, SD, standard deviation. Bold indicates significant p-values <0.05*.

### Successful PG Registrations and Comparison of Apnea/Hypopnea Index Measurements

**Figure 1:**
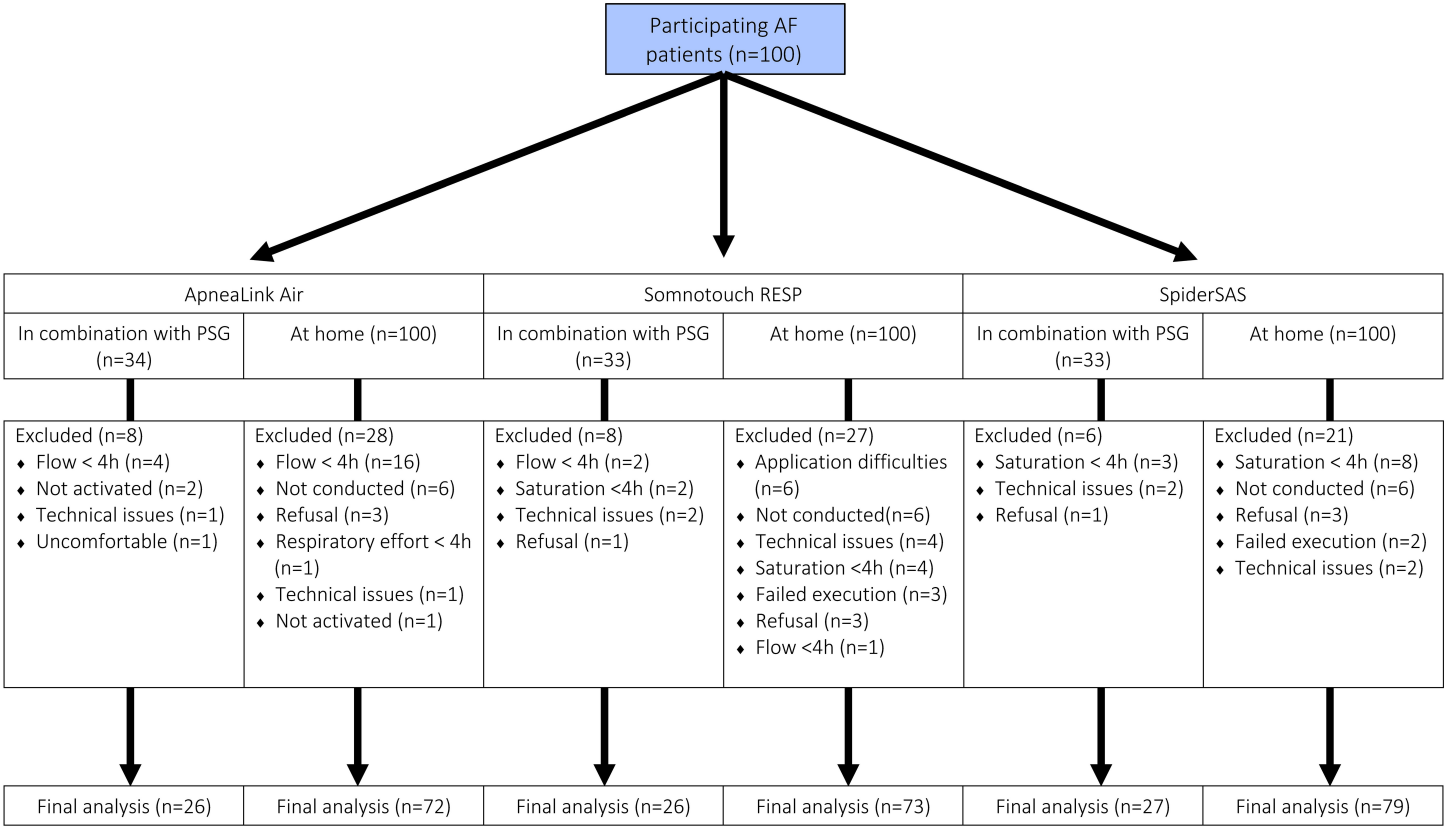
Successful PG registrations. AF, atrial fibrillation; PSG, polysomnography.

Successful and complete PG registration, i.e. ≥4 h of data on automated analysis, during PSG evaluation was obtained in 76.5, 78.8 and 81.8% of patients with the ALA, STR and SpS, respectively. Similarly for the PG executions at home, a success rate of 72.0, 73.0 and 79.0% was obtained for the ALA, STR and SpS, respectively (Figure 1). For the ALA, the main reason for data exclusion during PSG evaluation and execution at home, was <4 h of flow signal data in 20 registrations (14.9%) as analysed by the ResMED AirView software. For the STR and SpS, technical issues combined with application difficulties (9.0%) and <4 h of saturation signal data (8.3%) were the main reasons for data exclusion, respectively. When disregarding patient refusal and not conducting one of the PGs at home, actual successful PG execution was obtained in 79.1, 80.2 and 86.8% of patients with the ALA, STR and SpS, respectively.

When comparing the AHI of the PGs vs. the PSG measurements (both simultaneous and during separate nights), a significant difference (*p* <0.05) in the AHI was seen for the ALA and STR (Table 2). For the ALA, this is due to significant hypopnea underdetection. For the STR, hypopnea underdetection was also seen, but this PG also detected more apneas than PSG (*p* <0.01). On the other hand, the SpS detected more hypopneas compared to PSG, resulting in a significant Hypopnea Index difference between the SpS at home and PSG.

**Table 2:** Comparison of apnea/hypopnea indices by PSG and PGs.

	**PSG**	**ApneaLink Air**	**Δ**	**p**	**PSG**	**Somnotouch RESP**	**Δ**	**p**	**PSG**	**SpiderSAS**	**Δ**	**p**
	**Same night (*****n*** **=** **26)**				**Same night (*****n*** **=** **26)**				**Same night (*****n*** **=** **27)**			
AHI (events/h)	21.7 ± 14.9	14.9 ± 11.3	6.8 ± 7.4	<0.001	31.7 ± 23.4	25.8 ± 17.0	5.8 ± 9.8	0.007	25.9 ± 20.5	27.7 ± 12.7	−1.8 ± 12.3	0.343
AI (events/h)	4.5 ± 8.7	4.3 ± 6.1	0.2 ± 4.1	0.747	9.1 ± 13.4	12.8 ± 12.1	−3.7 ± 7.5	0.008	6.4 ± 12.1	6.0 ± 6.3	0.5 ± 9.0	0.211
HI (events/h)	17.1 ± 10.1	10.5 ± 6.7	6.6 ± 7.3	<0.001	22.6 ± 15.6	13.0 ± 7.8	9.6 ± 10.4	<0.001	19.4 ± 15.1	20.7 ± 11.0	−1.2 ± 11.7	0.374
	**Separate nights (*****n*** **=** **72)**				**Separate nights (*****n*** **=** **73)**				**Separate nights (*****n*** **=** **79)**			
AHI (events/h)	25.3 ± 18.9	16.9 ± 12.0	8.4 ± 14.0	<0.001	27.5 ± 20.6	21.4 ± 13.8	6.1 ± 17.1	0.028	27.2 ± 20.3	28.8 ± 12.	−1.6 ± 17.0	0.154
AI (events/h)	5.6 ± 9.6	4.6 ± 6.3	1.1 ± 7.8	0.431	6.2 ± 11.0	10.5 ± 10.3	−4.3 ± 10.4	<0.001	6.6 ± 11.0	4.8 ± 6.0	1.9 ± 8.8	0.359
HI (events/h)	19.6 ± 14.0	12.3 ± 7.9	7.3 ± 10.5	<0.001	21.3 ± 16.0	10.9 ± 9.0	10.4 ± 13.5	<0.001	20.5 ± 15.7	22.7 ± 10.8	−2.2 ± 15.2	0.039

*All values are represented as Mean and Standard Deviation. PSG, polysomnography; AHI, apnea-hypopnea index; AI, apnea index; HI, hypopnea index*.

The comparison between the PG measurements at the sleep clinic and at home, showed absolute AHI differences of 0.2, 2.9 and −0.5 for the ALA, STR and SpS, respectively. Intraindividual Night-to-Night Variability (NtNV) categorical change (at AHI cut-off of 15 events/h) was seen for 13.0, 36.8 and 9.5% of patients for the ALA, STR and SpS, respectively in these small samples (Supplementary Table 2).

### Performance of the Different PGs in Predicting OSA Severity in AF Patients

Table 3 shows the sensitivity, specificity, PPV, NPV, AUC and Cohen's kappa for the ALA, STR and SpS, categorised by the different AHI thresholds by PSG, by PG during PSG evaluation and by PG at home. For detecting at least mild OSA (AHI ≥ 5 events/h), all PGs had sensitivity values >90%. The SpS was too sensitive: it detected an AHI ≥ 5 for all patients resulting in zero specificity. For patients with an AHI ≥ 15 events/h, a sensitivity of >90% was only reached for the SpS, but specificity remained low. The ALA and STR had specificity values >90% for detecting severe OSA (AHI > 30 events/h). Nevertheless, sensitivity remained low for all PGs in this category. The Cohen's Kappa coefficient reached values >0.40 for the ALA and STR for detecting at least mild OSA and for the STR for detecting severe OSA, reflecting rather moderate agreement with the gold standard PSG in these categories.

**Table 3:** Performance of the PGs in predicting OSA severity in AF patients.

**PSG Cut-off**		**ApneaLink Air**		**Somnotouch RESP**		**SpiderSAS**	
		**During PSG**	**At home**	**During PSG**	**At home**	**During PSG**	**At home**
		**(*n* = 26)**	**(*n* = 72)**	**(*n* = 26)**	**(*n* = 73)**	**(*n* = 27)**	**(*n* = 79)**
AHI ≥ 5	Sensitivity (%)	90.5	93.8	95.7	97.0	100.0	100
	Specificity (%)	100.0	50	66.7	42.9	/	0.0
	PPV (%)	100.0	93.8	95.7	94.1	100.0	88.6
	NPV (%)	71.4	50.0	66.7	60.0	/	/
	AUC (95% CI)	0.962 (0.885–1.000)	0.854 (0.728–0.979)	0.928 (0.811–1.000)	0.834 (0.670–0.998)	/	0.752 (0.605–0.900)
	κ	0.79	0.44	0.62	0.46	/	/
AHI ≥ 15	Sensitivity (%)	57.9	62.5	94.4	74.5	100.0	98.2
	Specificity (%)	100.0	79.2	83.3	63.6	25.0	25.0
	PPV (%)	100.0	85.7	94.4	82.6	62.5	75.0
	NPV (%)	46.7	51.4	62.5	51.9	100.0	85.7
	AUC (95% CI)	0.940 (0.845–1.000)	0.802 (0.694–0.910)	0.917 (0.785–1.000)	0.772 (0.657–0.888)	0.853 (0.710–0.996)	0.803 (0.702–0.903)
	κ	0.43	0.37	0.61	0.36	0.27	0.29
AHI > 30	Sensitivity (%)	75.0	31.8	84.6	45.8	90.0	60.7
	Specificity (%)	100.0	94.0	100.0	93.9	82.4	74.5
	PPV (%)	100.0	70.0	100.0	78.6	75.0	56.7
	NPV (%)	88.5	75.8	86.7	78.0	93.3	77.6
	AUC (95% CI)	0.852 (0.592–1.000)	0.758 (0.642–0.873)	0.976 (0.924–1.000)	0.780 (0.664–0.897)	0.953 (0.881–1.000)	0.772 (0.668–0.876)
	κ	0.84	0.31	0.85	0.44	0.70	0.35

*AHI, apnea-hypopnea index; PPV, positive predictive value; NPV, negative predictive value; AUC, area under the curve; CI, confidence interval; κ, Cohen's Kappa; “dark” and “light” grey boxes reflect very good and good values respectively for sensitivity, specificity, PPV and NPV. For AUC and Cohen's Kappa (κ), “dark” and “light” grey boxes reflect excellent and good values, respectively*.

**Figure 2:**
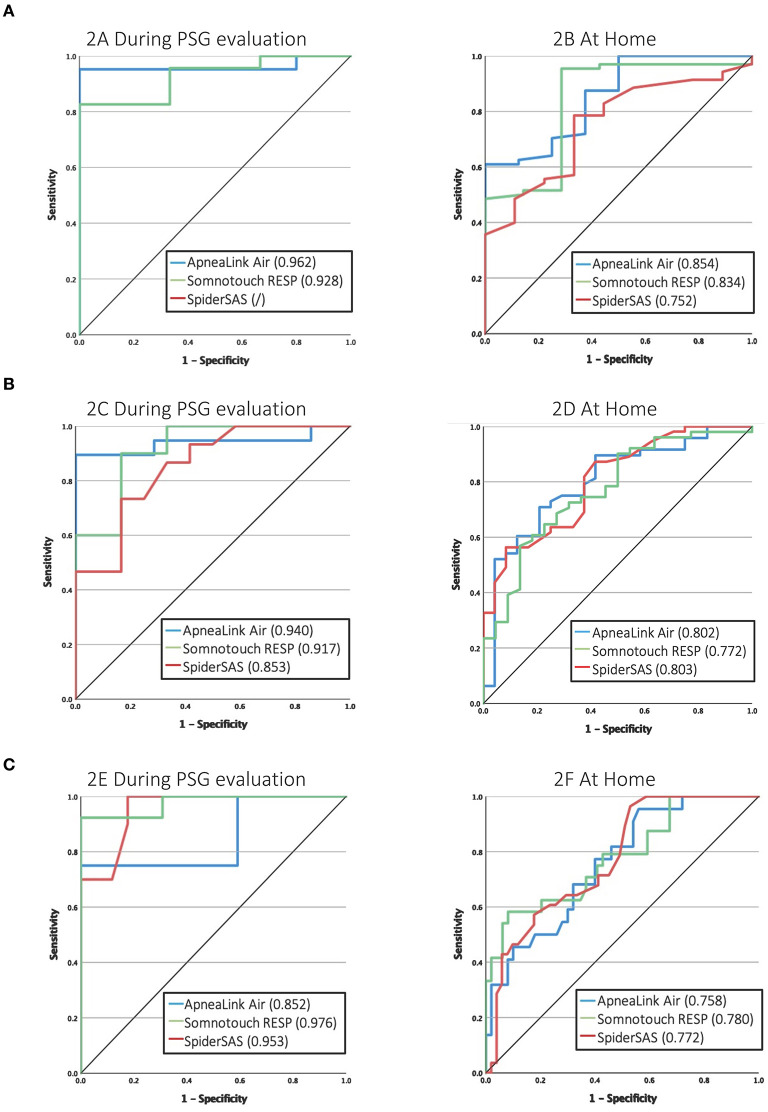
ROC curves in predicting OSA severity in AF patients. **(A)** Mild OSA (AHI ≥ 5). **(B)** Moderate OSA (AHI ≥ 15). **(C)** Severe OSA (AHI ≥ 30). OSA, obstructive sleep apnea; AHI, apnea-hypopnea index.

**Figure 3:**
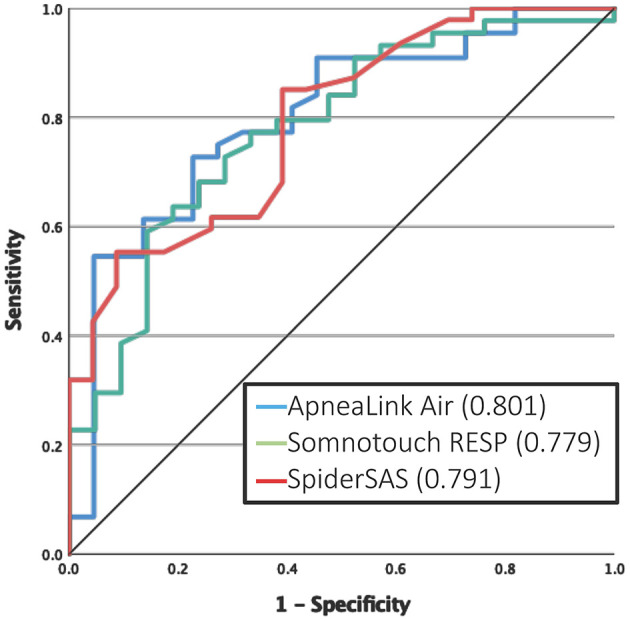
ROC curves in predicting clinical relevant OSA in non-permanent AF patients.

For the detection of at least mild OSA, the ALA and STR had a good AUC of 0.854 and 0.834, respectively (Figure 2B). At the threshold of ≥15 events/h, the ALA and SpS had good discriminative potential with an AUC of 0.802 and 0.803, respectively (Figure 2D). For severe OSA (AHI >30 events/h), none of the PGs reached an AUC value >0.80 (Figure 2F). A subanalysis was performed for predicting at least moderate OSA in only non-permanent AF patients, which resulted in an AUC of 0.801, 0.779 and 0.791 for the ALA, STR and SpS, respectively (Figure 3).

### Determination of Appropriate PG Cut-off Values for Detection of Clinically Relevant OSA

Coordinates of the AHI cut-off values with their corresponding sensitivity, specificity and Youden's J Index for the ALA, STR and SpS can be found in Supplementary Tables 3–5. For all PGs, cut-off values were selected for the best discriminating ability to rule in or rule out clinically relevant OSA (AHI ≥ 15) combined with the optimal Youden's J Index. Optimal sensitivity values were obtained for AHI cut-off values 7.0, 10.3 and 19.5 events/h for the ALA, STR and SpS, respectively. Similarly, optimal specificity values were obtained for AHI cut-off values 19.9, 19.9 and 29.5 events/h for the ALA, STR and SpS, respectively (Table 4).

**Table 4:** Selected cut-off values of the different PGs for predicting clinical relevant OSA based on home measurements.

	**Sensitivity (%)**	**Specificity (%)**	**PPV (%)**	**NPV (%)**	**Youden's J Index**	**Accuracy**
**ApneaLink Air (*****n*** **=** **72)**						
AHI = 7.0 events/h	89.6	58.3	81.1	73.7	0.479	0.79
AHI = 19.9 events/h	52.1	95.8	96.2	50.0	0.479	0.67
**Somnotouch RESP (*****n*** **=** **73)**						
AHI = 10.3 events/h	90.2	50.0	80.7	68.8	0.402	0.78
AHI = 19.9 events/h	56.9	86.4	90.6	46.3	0.433	0.66
**SpiderSAS (*****n*** **=** **79)**						
AHI = 19.5 events/h	87.3	58.3	82.8	66.7	0.460	0.78
AHI = 29.5 events/h	56.4	91.7	93.9	47.8	0.480	0.67

*AHI, apnea-hypopnea index; PPV, positive predictive value; NPV, negative predictive value*.

### Convenience of the PG Devices

An overall comfort score of 8.25 ± 1.42, 7.97 ± 1.39, 6.63 ± 1.90 and 5.97 ± 2.32 (out of 10) was given for the ALA, SpS, STR and PSG, respectively (*p* <0.05 for all PGs vs. PSG, ALA vs. STR and SpS vs. STR). Self-application of the PGs at home was found easier for the ALA than the SpS and the STR (*p* <0.05). In addition, the study patients indicated that their sleep was less disturbed by the ALA, SpS and STR compared with PSG (*p*-values of 0.01, 0.01 and 0.09 respectively).

## Discussion

This is the first study evaluating and validating the use of different (cardio)respiratory PG devices, combined with an automated algorithm for AHI determination, as OSA detection tools in AF patients, compared with PSG as gold standard. Our results indicate that the tested PGs combined with their automated algorithm can be used for detecting clinically relevant OSA (i.e., AHI ≥ 15 events/h) in AF patients, especially when appropriate cut-off values are chosen for each device, and for the screening scenario at hand.

### Screening for OSA in AF Patients and Current Screening Tools

As mentioned before, OSA is highly underdiagnosed in the AF population, despite a prevalence of up to 86% in AF patients recruited in a community cardiology clinic (14). Our study is in line with this finding, although our study patients had a higher pretest probability. Nevertheless, the high prevalence of OSA and the recommendation for optimal OSA treatment in the general management of AF patients makes screening for OSA useful in this population. Several OSA screening tools already exist but are not properly validated in the AF population. Our research group has tested validated and commonly used OSA screening questionnaires and scoring systems in the same AF cohort. For the Epworth Sleepiness Scale, Berlin Questionnaire, Sleep Apnea Clinical Score (SACS), NoSAS, OSA50, STOP-Bang and MOODS, the AUCs were 0.532, 0.626, 0.704, 0.712, 0.686, 0.673 and 0.655 respectively. Thus these screening tools are lacking sufficient performance for testing for clinical relevant OSA (15). Although the 2020 ESC AF Guidelines do not recommend a particular OSA screening tool in AF patients, several reviews have proposed OSA screening pathways in which PG evaluation may play a role (4, 6). Our study may underpin such pathways since it provides data on the reliability and practical implementation of PG in the detection of OSA in AF patients in clinical practise, along with data on patients' experience with different PG devices.

### Validity of PG Evaluation Combined With Automated Analysis

Apnea/hypopnea (AH) indices of PG cannot simply substitute for the same index by PSG. Firstly, a lower AHI was seen for the ALA and STR compared to PSG, driven by a lower hypopnea detection. This is due to the fact that PG cannot detect arousals which also contributes to the hypopnea definition according to the 2012 AASM guidelines (13). Consequently and confirmed by this study, AF patients investigated by the ALA and STR are likely to have a 30% lower AHI on average, compared with PSG (16). Surprisingly, the SpS with its automated analysis barely detected a difference of apneas and hypopneas and thus the AHI also remained equal to that of PSG. In fact, this questions the reliability of the AHI evaluation of the SpS combined with its automated algorithm. These differences in AH indices are seen for the different PSG-PG comparisons (i.e., PSG vs. PG in-hospital or at home) for which the (more numerous) PG measurements at home can be a good representation of the in-hospital PG measurements.

Secondly, analysis of the paired PG evaluations showed only minor absolute AHI differences for the 3 PGs, although the intraindividual NtNV categorical change (at AHI cut-off of 15 events/h) varied between 9.5 and 36.8%. These findings are in line with a recent meta-analysis, in which the absolute AHI NtNV for PGs was calculated to be on average −0.2 events/h (95% CI −1.19–0.79), but also a high intraindividual NtNV categorical change was identified (17). The latter reflects the ongoing debate of a single-night sleep evaluation for OSA diagnosing due to physiological night-to-night variations, but this is out of the scope of this study.

Thirdly, for detecting clinically relevant OSA (i.e., AHI ≥ 15), the ALA and SpS performed well with an AUC > 0.80 (at home) of which the ALA had a higher measurement agreement than the SpS, reflected by the Cohen's Kappa. When comparing these results with literature, caution is warranted since other studies often used other methods or definitions for AHI scoring or other gold standards for OSA diagnosis. For level 3 sleep tests in general, a meta-analysis indicated that the AUC for detecting at least moderate OSA ranged between 0.87–0.99 and 0.79–0.97 compared with simultaneous or separate PSG evaluation, respectively, although PG recordings were manually reviewed in the studies included (18). Regarding the ALA with PSG comparison, data is only publicly available for the previous generation of the ALA, i.e., the ApneaLink Plus (ALP), which lacks the body-position sensor compared with the ALA. Their analysis software, including the automated algorithm, is the same. Cho and Kim compared the ALP (used with automated AASM analysis) with PSG in 149 patients referred for habitual snoring or witnessed apneas to the sleep centre (19). The AUC of the ALP for an AHI ≥ 15 was 0.924 and 0.845 during simultaneous and separate PSG evaluation, respectively, and thus comparable with our results (19).

### Other Factors Related to the Usability of PG as an OSA Detection Tool

Our study reports an average success rate of 82% for PG registrations at home. In general, for level 3 sleep testing devices, as evaluated in this study, a meta-analysis reported a (technical) success rate of 89.75% compared with 99.56% in patients evaluated by PSG (18). Importantly, the mean age of patients of the included studies was 50.8 years which could influence the higher success rates. In more recent studies, success rates vary widely between 69.8 and 96.3% in patients evaluated by the ALP. (19, 20) For this specific PG, <4 h of nasal flow or oximetry signal was also the main reason for data exclusion (20). An average success rate of 82% for PG registrations at home means that a second PG evaluation or direct PSG examination may be necessary for a part of the AF population.

As CPAP treatment in Belgium (as in other countries) is only reimbursed for clinically relevant OSA (AHI ≥ 15) (12), we determined optimised AHI cut-offs for each PG for optimal sensitivity or specificity to detect this patient group (Table 4; Figure 2; Supplementary Tables 3–5). For the ALA and STR these values lie in the same range, but for the SpS both cut-offs needed to be about 10 units higher. As discussed, this is explained by the contradictory high(er) detection of hypopneas by the SpS.

The three PGs scored significantly better than PSG in terms of convenience, i.e., better sleep quality and less troublesomeness caused by the PGs' equipment. When comparing the tested PG with each other, the ALA was easier to attach than the SpS and STR. This can be explained by the simplified attachment of the ALA with fewer channels. One prior study also investigated the convenience of the ALA, in which 98.5% of patients were positive about its use, 67.6% assessed the comfort very well and 32.4% only mentioned minor comments regarding the nasal cannula or oximeter (21).

Finally, the ease of administering these PGs and the use of their software for clinicians should also be mentioned. A good manual for the patient can be time-saving, and a software that can generate a report within 5 min so that results and possibly referral for PSG evaluation can be discussed instantly during a patients' out-patient clinic visit or during a hospitalisation are of added value. These features are present for both the ALA and STR with their corresponding software.

### Clinical Pathway Proposal for OSA Detection in AF Patients

As our cohort included 13 patients with permanent AF, i.e., patients not considered anymore for rhythm control therapy and hence OSA screening, the validity of the PGs was reassessed excluding these patients: the new AUCs for detecting clinically relevant OSA showed only minor changes (Figure 3). Although these permanent AF patients cannot benefit from improved rhythm control anymore, in case of OSA detection, treatment with CPAP therapy can still improve the quality of life and OSA-related symptoms in these patients.

Based on our results, a proposed detection strategy could consist of applying a PG device to all AF patients in whom rhythm control is pursued, regardless of apnea symptoms. If the PG evaluation produces reliable tracings, the automated algorithm software program can analyse the data to determine the AHI. The treating cardiologist or AF-clinic coordinator can do this read-out and interpretation. As CPAP treatment of OSA in AF patients is currently only associated with better rhythm and symptom control but not (yet) with clinical outcomes (such as mortality, stroke,…), a PG AHI cut-off associated with high specificity would be preferable (Table 4) (3). In this way, only limited false positives would be detected, avoiding redundant, burdensome and expensive overnight PSG evaluations with already long waiting lists. On the other hand, higher false negative results can be expected for which an additional PG evaluation or advice from a somnologist may be the next step in the case of high clinical suspicion (e.g., persistent daytime sleepiness, frequent loud snoring, witnessed apneas). If future studies would indicate that CPAP treatment also positively impacts hard clinical outcomes, the appropriate cut-off values have to be revised. This proposed pathway definitely needs proper prospective evaluation in selected AF patients seen at the Cardiology department, including cost-effectiveness assessment of CPAP initiation.

The strength of our study is the comparison of the PGs with PSG as gold standard for the diagnosis of OSA in which the simultaneous and separately executed PG evaluations provide direct insights in the detection of sleep-disordered breathing events by the automated analysis of PG recordings and the influence of NtNV. Additionally, our included patients had a mean age of 64 years for which the success rate of PG execution better reflects ‘real-world' clinical AF practise than prior PG studies.

### Limitations

The included AF patients, referred for a diagnostic PSG, may not be representative for the selected AF population that one would like to screen in daily clinical practise (i.e., symptomatic AF patients treated with or undergoing antiarrhythmic therapy), although 73.6% of the non-permanent AF patients had a mEHRA score ≥ 2a (i.e., were symptomatic). The scoring of the PSG was performed in two different centres, which may include interscorer variability. Lastly, this study design was rather intensive (4 nights of PG testing), which could influence the success rates for each PG at home.

## Conclusion

This validation study underscores that PG combined with an automated algorithm for AHI determination and appropriately selected AHI cut-offs can be used as a reliable OSA detection tool in an AF clinic. As implementation of the current AF guidelines, the clinician can easily screen AF patients using a PG device and refer for confirmatory PSG evaluation (and CPAP treatment) to ensure proper addressing of OSA as a modifiable risk factor.

## Data Availability Statement

The raw data supporting the conclusions of this article will be made available by the authors, without undue reservation.

## Ethics Statement

The studies involving human participants were reviewed and approved by University of Antwerp/Antwerp University Hospital and Jessa Hospital Ethics Committees. The patients/participants provided their written informed consent to participate in this study.

## Author Contributions

MD, JV, HH, LD, KW, and PD contributed to conception and design of the study. MD, LK, and LD collected the data and organised the database. MD performed the statistical analysis and wrote the first draft of the manuscript. All authors contributed to manuscript revision, read, and approved the submitted version.

## Funding

This study was supported by the Antwerp University Hospital Cardiology Research Fund, and is part of the Limburg Clinical Research Center, supported by the foundation Limburg Sterk Merk, province of Limburg, Flemish government, Hasselt University, Ziekenhuis Oost-Limburg and Jessa Hospital.

## Conflict of Interest

HH and LD did not receive any personal honoraria; they received unconditional research support through Hasselt University or University of Antwerp from Bayer, Daiichi-Sankyo, Boehringer-Ingelheim, Bracco Imaging Europe, Medtronic, Boston-Scientific, Biotronik, and St. Jude Medical. JV reports grants from SomnoMed, AirLiquide, Vivisol, Mediq Tefa, Medidis, Heinen-Löwenstein, OSG, Bioprojet, Desitin, Philips and ResMed outside the submitted work. The remaining authors declare that the research was conducted in the absence of any commercial or financial relationships that could be construed as a potential conflict of interest.

## Publisher's Note

All claims expressed in this article are solely those of the authors and do not necessarily represent those of their affiliated organizations, or those of the publisher, the editors and the reviewers. Any product that may be evaluated in this article, or claim that may be made by its manufacturer, is not guaranteed or endorsed by the publisher.
