# Supplementary material for: Cardiorespiratory Polygraphy for Detection of Obstructive Sleep Apnea in Patients With Atrial Fibrillation
Source: Front Cardiovasc Med. 2021 Nov 30;8:758548. doi: 10.3389/fcvm.2021.758548 (PMC8669303; doi:10.3389/fcvm.2021.758548)
Supplement: Supplementary file 1 [file Data_Sheet_1.docx]

# Supplement

## **Supplementary annex 1: Comfort Questionnaire**

** The first two questions were not applicable when evaluating the polysomnographic examination.*

1. How clear were the instructions for this device?*

| 0 | 1 | 2 | 3 | 4 | 5 | 6 | 7 | 8 | 9 | 10 |
| --- | --- | --- | --- | --- | --- | --- | --- | --- | --- | --- |

Unclear Very clear

1. Was this device easy to attach?*

| 0 | 1 | 2 | 3 | 4 | 5 | 6 | 7 | 8 | 9 | 10 |
| --- | --- | --- | --- | --- | --- | --- | --- | --- | --- | --- |

Very difficult Very easy

1. Did you experience any discomfort during your sleep due to this device?

| 0 | 1 | 2 | 3 | 4 | 5 | 6 | 7 | 8 | 9 | 10 |
| --- | --- | --- | --- | --- | --- | --- | --- | --- | --- | --- |

No discomfort Very uncomfortable

1. How did you sleep last night?

| 0 | 1 | 2 | 3 | 4 | 5 | 6 | 7 | 8 | 9 | 10 |
| --- | --- | --- | --- | --- | --- | --- | --- | --- | --- | --- |

Restless Peaceful

1. In general, what score would you give based on (the difficulty of attachment and) comfort of this device?

| 0 | 1 | 2 | 3 | 4 | 5 | 6 | 7 | 8 | 9 | 10 |
| --- | --- | --- | --- | --- | --- | --- | --- | --- | --- | --- |

Bad Good

1. Did you experience any symptoms last night that may indicate atrial fibrillation (palpitations, irregular pulse, shortness of breath, chest pain)?

| 0 | 1 | 2 | 3 | 4 | 5 | 6 | 7 | 8 | 9 | 10 |
| --- | --- | --- | --- | --- | --- | --- | --- | --- | --- | --- |

No symptoms Severe symptoms

1. Did this device interfere with your sleep?

| 0 | 1 | 2 | 3 | 4 | 5 | 6 | 7 | 8 | 9 | 10 |
| --- | --- | --- | --- | --- | --- | --- | --- | --- | --- | --- |

No interference Severe interference

1. What score would you give to your sleep last night?

| 0 | 1 | 2 | 3 | 4 | 5 | 6 | 7 | 8 | 9 | 10 |
| --- | --- | --- | --- | --- | --- | --- | --- | --- | --- | --- |

Poor Good

Other remarks or suggestions:

| … |
| --- |

## **Supplementary table 1:** **Specifications of the tested polygraphs**

| Measurements |  | ApneaLink Air (ResMED) | Somnotouch RESP (SOMNOmedics) | SpiderSAS (MicroPort) |
| --- | --- | --- | --- | --- |
|  |  | 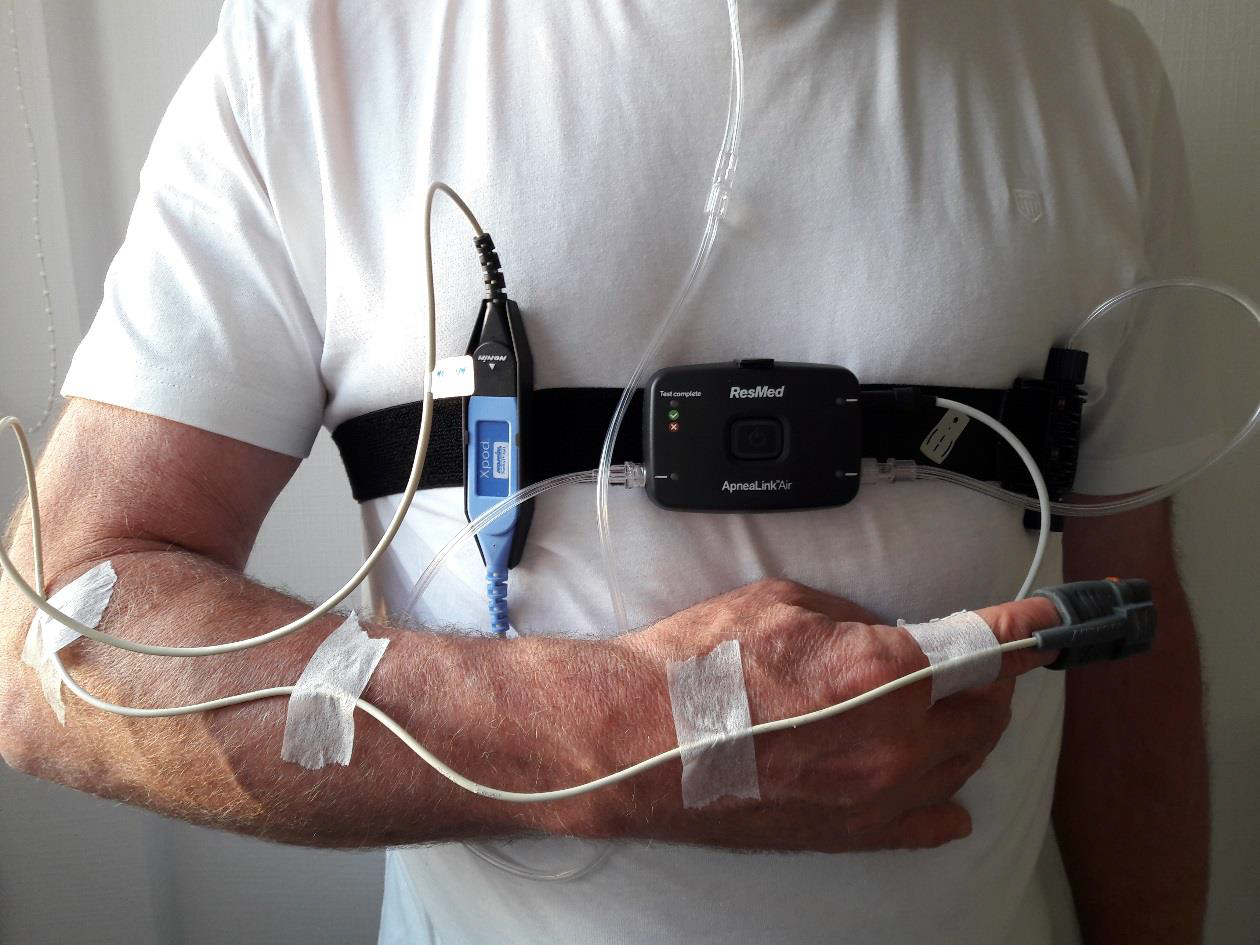 | 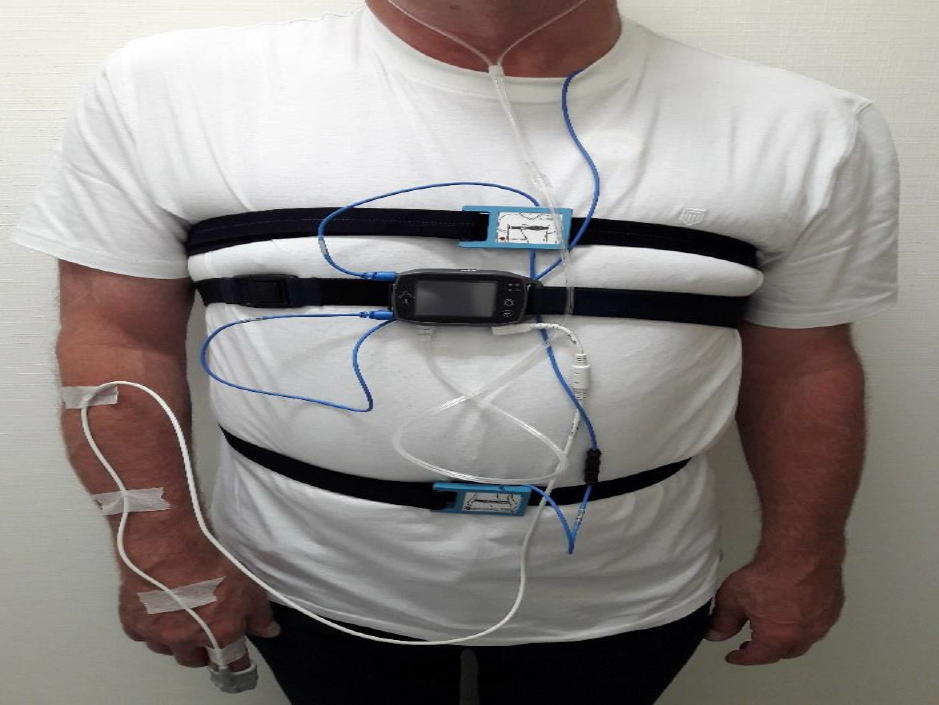 | 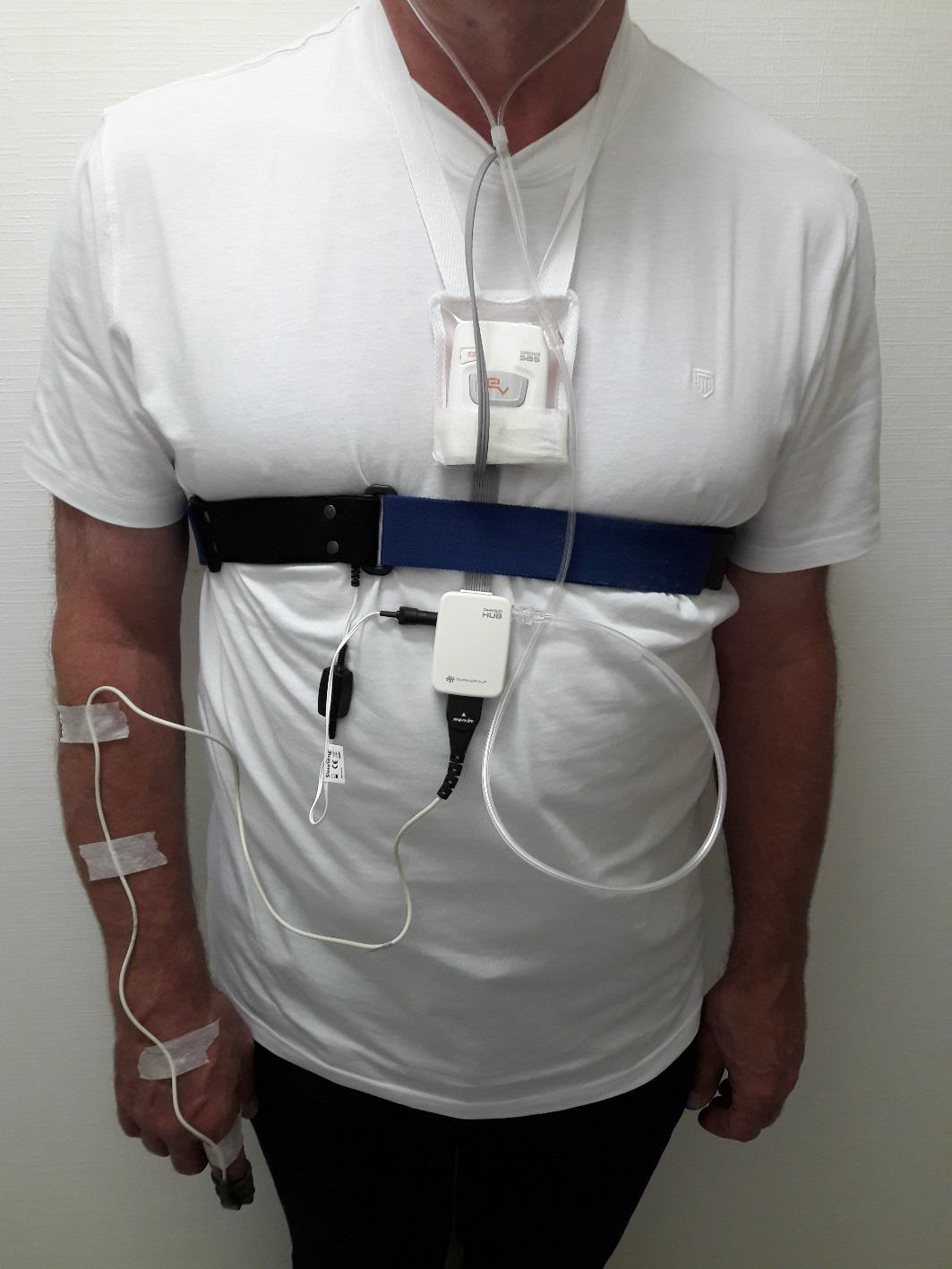 |
| Electrocardiography |  | / | ECG (3 chest electrodes) | ECG (3 chest electrodes) |
| Pulse rate |  | PPG (pulse oximeter) | PPG (pulse oximeter) | PPG (pulse oximeter) |
| Blood oxygen saturation |  | Oximeter finger (SpO2) | Oximeter finger (SpO2) | Oximeter finger (SpO2) |
| Nasal pressure |  | Nasal cannula pressure sensor | Nasal cannula pressure sensor | Nasal cannula pressure sensor |
| Thoracic respiratory effort |  | Thoracic belt sensor (Piezo effort sensor) | Thoracic belt sensor (RIP) | Thoracic belt (Piezo effort sensor) |
| Abdominal respiratory effort |  | / | Abdominal belt sensor (RIP) | / |
| Snoring events |  | Snore sensor (nasal pressure) | Snore sensor (nasal pressure) | / |
| Movement |  | Movement sensor | Movement sensor | 3D Accelerometer |
| Body position |  | Body position sensor | Body position sensor | 3D Accelerometer |
| Activation |  | Manually | Automatically | Automatically |
| Software |  | AirView version 4.7.2-21.0.0 | DOMINO light version 1.5.0 | SYNESCOPE Version 3.30 |

## **Supplementary table 2: Comparison of Apnea/Hypopnea indices by PG measurements at the hospital/home**

|  | ApneaLink Air (n=23) | | | | Somnotouch RESP (n=19) | | | | SpiderSAS (n=21) | | | |
| --- | --- | --- | --- | --- | --- | --- | --- | --- | --- | --- | --- | --- |
|  | Hospital | Home | △ | p | Hospital | Home | △ | p | Hospital | Home | △ |  |
| AHI (events/h) | 13.4 ± 10.1 | 13.1 ± 9.6 | 0.2 ± 6.1 | 0.715 | 25.3 ± 19.0 | 22.4 ± 13.0 | 2.9 ± 14.8 | 0.444 | 28.0 ± 10.3 | 28.5 ± 11.9 | -0.5 ± 9.3 | 0.577 |
| AI (events/h) | 3.5 ± 4.1 | 2.7 ± 3.3 | 0.8 ± 3.0 | 0.191 | 12.7 ± 13.7 | 10.2 ± 9.2 | 2.5 ± 10.3 | 0.365 | 7.1 ± 6.8 | 5.6 ± 6.9 | 1.6 ± 5.4 | 0.175 |
| HI (events/h) | 9.9 ± 6.8 | 10.5 ± 7.5 | -0.6 ± 4.8 | 0.584 | 12.6 ± 8.6 | 12.2 ± 7.9 | 0.4 ± 9.1 | 0.763 | 19.5 ± 7.0 | 21.7 ± 8.3 | -2.2 ± 8.4 | 0.158 |
|  |  |  |  |  |  |  |  |  |  |  |  |  |

All values are represented as Mean and Standard Deviation. PSG: Polysomnography, AHI: Apnea-Hypopnea index, AI: Apnea Index, HI: Hypopnea Index

|  | | ApneaLink Air (n=23) | | Somnotouch RESP (n=19) | | SpiderSAS (n=21) | |
| --- | --- | --- | --- | --- | --- | --- | --- |
|  |  | Hospital | | Hospital | | Hospital | |
|  |  | AHI ≥ 15 (n) | AHI < 15 (n) | AHI ≥ 15 (n) | AHI < 15 (n) | AHI ≥ 15 (n) | AHI < 15 (n) |
| Home | AHI ≥ 15 (n) | 8 | 2 | 9 | 4 | 18 | 1 |
|  | AHI < 15 (n) | 1 | 12 | 3 | 3 | 1 | 1 |
|  |  |  |  |  |  |  |  |
| Intraindividual NtNV categorical change (%) | | 13.0 | | 36.8 | | 9.5 | |

NtNV=Night-to-Night Variability, AHI: Apnea-Hypopnea index (events/h)

## **Supplementary table 3: Coordinates of the ROC-curve of the ApneaLink Air in predicting clinically relevant OSA**

| AHI cut-off (events/h) | Sensitivity (%) | Specificity (%) | Youden’s J Index |
| --- | --- | --- | --- |
| 0.7 | 100.0 | 0.0 | 0.000 |
| 1.8 | 100.0 | 4.2 | 0.042 |
| 2.55 | 100.0 | 8.3 | 0.083 |
| 3.35 | 100.0 | 12.5 | 0.125 |
| 3.9 | 100.0 | 16.7 | 0.167 |
| 4.4 | 97.9 | 16.7 | 0.146 |
| 4.6 | 95.8 | 16.7 | 0.125 |
| 4.75 | 95.8 | 20.8 | 0.166 |
| 4.9 | 95.8 | 25.0 | 0.208 |
| 5.05 | 93.8 | 25.0 | 0.188 |
| 5.2 | 91.7 | 25.0 | 0.167 |
| 5.5 | 91.7 | 29.2 | 0.209 |
| 5.8 | 91.7 | 33.3 | 0.250 |
| 5.95 | 91.7 | 37.5 | 0.292 |
| 6.1 | 91.7 | 41.7 | 0.334 |
| 6.4 | 89.6 | 41.7 | 0.313 |
| 6.7 | 89.6 | 45.8 | 0.354 |
| 6.85 | 89.6 | 54.2 | 0.438 |
| 7.0 | 89.6 | 58.3 | 0.479 |
| 7.75 | 85.4 | 58.3 | 0.437 |
| 8.5 | 83.3 | 58.3 | 0.416 |
| 8.85 | 81.3 | 58.3 | 0.396 |
| 9.95 | 79.2 | 62.5 | 0.417 |
| 11.05 | 77.1 | 62.5 | 0.396 |
| 11.55 | 75.0 | 62.5 | 0.375 |
| 11.85 | 75.0 | 66.7 | 0.417 |
| 12.35 | 75.0 | 70.8 | 0.458 |
| 13.05 | 72.9 | 75.0 | 0.479 |
| 13.35 | 70.8 | 75.0 | 0.458 |
| 13.5 | 70.8 | 79.2 | 0.500 |
| 13.8 | 68.8 | 79.2 | 0.480 |
| 14.3 | 66.7 | 79.2 | 0.459 |
| 14.7 | 64.6 | 79.2 | 0.438 |
| 15.15 | 62.5 | 79.2 | 0.417 |
| 15.65 | 60.4 | 79.2 | 0.396 |
| 15.95 | 60.4 | 83.3 | 0.437 |
| 16.75 | 60.4 | 87.5 | 0.479 |
| 17.65 | 58.3 | 87.5 | 0.458 |
| 17.95 | 56.3 | 87.5 | 0.438 |
| 18.45 | 54.2 | 87.5 | 0.417 |
| 19.1 | 54.2 | 91.7 | 0.459 |
| 19.5 | 52.1 | 91.7 | 0.438 |
| 19.85 | 52.1 | 95.8 | 0.479 |
| 20.35 | 47.9 | 95.8 | 0.437 |
| 20.9 | 45.8 | 95.8 | 0.416 |
| 21.2 | 43.8 | 95.8 | 0.396 |
| 21.4 | 41.7 | 95.8 | 0.375 |
| 21.55 | 39.6 | 95.8 | 0.354 |
| 21.7 | 35.4 | 95.8 | 0.312 |
| 21.85 | 33.3 | 95.8 | 0.291 |
| 22.45 | 31.3 | 95.8 | 0.271 |
| 23.3 | 29.2 | 95.8 | 0.250 |
| 23.9 | 27.1 | 95.8 | 0.229 |
| 25.5 | 25.0 | 95.8 | 0.208 |
| 27 | 22.9 | 95.8 | 0.187 |
| 27.35 | 20.8 | 95.8 | 0.166 |
| 29.35 | 18.8 | 95.8 | 0.146 |
| 31.35 | 16.7 | 95.8 | 0.125 |
| 31.9 | 14.6 | 95.8 | 0.104 |
| 32.4 | 12.5 | 95.8 | 0.083 |
| 33.2 | 10.4 | 95.8 | 0.062 |
| 34.45 | 8.3 | 95.8 | 0.041 |
| 36.35 | 6.3 | 95.8 | 0.021 |
| 40.65 | 6.3 | 100.0 | 0.063 |
| 43.9 | 4.2 | 100.0 | 0.042 |
| 56.95 | 2.1 | 100.0 | 0.021 |
| 70.7 | 0.0 | 100.0 | 0.000 |

## **Supplementary table 4: Coordinates of the ROC-curve of the Somnotouch RESP in predicting clinically relevant OSA**

| AHI cut-off (events/h) | Sensitivity (%) | Specificity (%) | Youden’s J Index |
| --- | --- | --- | --- |
| 1.5 | 100.0 | 0.0 | 0 |
| 3.05 | 98.0 | 0.0 | -0.020 |
| 3.75 | 98.0 | 4.5 | 0.025 |
| 4.05 | 98.0 | 9.1 | 0.071 |
| 4.35 | 98.0 | 13.6 | 0.116 |
| 5.35 | 98.0 | 18.2 | 0.162 |
| 6.75 | 98.0 | 22.7 | 0.207 |
| 7.4 | 96.1 | 22.7 | 0.188 |
| 7.65 | 96.1 | 27.3 | 0.234 |
| 7.85 | 96.1 | 31.8 | 0.279 |
| 8.1 | 96.1 | 36.4 | 0.325 |
| 8.4 | 94.1 | 36.4 | 0.305 |
| 8.55 | 92.2 | 36.4 | 0.286 |
| 9.1 | 92.2 | 40.9 | 0.331 |
| 9.85 | 92.2 | 45.5 | 0.377 |
| 10.15 | 90.2 | 45.5 | 0.357 |
| 10.25 | 90.2 | 50.0 | 0.402 |
| 10.45 | 86.3 | 50.0 | 0.363 |
| 10.65 | 84.3 | 50.0 | 0.343 |
| 11.5 | 82.4 | 50.0 | 0.324 |
| 12.45 | 80.4 | 50.0 | 0.304 |
| 12.65 | 78.4 | 50.0 | 0.284 |
| 12.75 | 78.4 | 54.5 | 0.329 |
| 13.25 | 76.5 | 54.5 | 0.310 |
| 13.75 | 74.5 | 54.5 | 0.290 |
| 14.2 | 74.5 | 59.1 | 0.336 |
| 14.9 | 74.5 | 63.6 | 0.381 |
| 15.35 | 72.5 | 63.6 | 0.361 |
| 15.75 | 72.5 | 68.2 | 0.407 |
| 16.2 | 70.6 | 68.2 | 0.388 |
| 16.65 | 68.6 | 72.7 | 0.413 |
| 17.3 | 66.7 | 72.7 | 0.394 |
| 17.9 | 64.7 | 72.7 | 0.374 |
| 18.25 | 64.7 | 77.3 | 0.420 |
| 18.55 | 62.7 | 77.3 | 0.400 |
| 18.8 | 60.8 | 77.3 | 0.381 |
| 19 | 60.8 | 81.8 | 0.426 |
| 19.35 | 58.8 | 81.8 | 0.406 |
| 19.9 | 56.9 | 86.4 | 0.433 |
| 20.7 | 54.9 | 86.4 | 0.413 |
| 21.5 | 52.9 | 86.4 | 0.393 |
| 21.85 | 51,0 | 86.4 | 0.374 |
| 22.1 | 49,0 | 86.4 | 0.354 |
| 22.6 | 43.1 | 86.4 | 0.295 |
| 23 | 41.2 | 86.4 | 0.276 |
| 23.5 | 39.2 | 90.9 | 0.301 |
| 24.95 | 37.3 | 90.9 | 0.282 |
| 26.9 | 35.3 | 90.9 | 0.262 |
| 28.05 | 33.3 | 90.9 | 0.242 |
| 28.4 | 31.4 | 90.9 | 0.223 |
| 28.8 | 29.4 | 90.9 | 0.203 |
| 29.5 | 29.4 | 95.5 | 0.249 |
| 29.95 | 27.5 | 95.5 | 0.230 |
| 30.35 | 25.5 | 95.5 | 0.210 |
| 32.65 | 23.5 | 95.5 | 0.190 |
| 35 | 23.5 | 100.0 | 0.235 |
| 35.45 | 21.6 | 100.0 | 0.216 |
| 37.75 | 19.6 | 100.0 | 0.196 |
| 40.45 | 17.6 | 100.0 | 0.176 |
| 41.6 | 15.7 | 100.0 | 0.157 |
| 44.15 | 13.7 | 100.0 | 0.137 |
| 46.05 | 11.8 | 100.0 | 0.118 |
| 47.8 | 9.8 | 100.0 | 0.098 |
| 51.85 | 7.8 | 100.0 | 0.078 |
| 54.5 | 5.9 | 100.0 | 0.060 |
| 55.6 | 3.9 | 100.0 | 0.040 |
| 57.55 | 2.0 | 100.0 | 0.020 |
| 59.7 | 0.0 | 100.0 | 0.000 |

## **Supplementary table 5: Coordinates of the ROC-curve of the SpiderSAS in predicting clinically relevant OSA**

| AHI cut-off (events/h) | Sensitivity (%) | Specificity (%) | Youden’s J Index |
| --- | --- | --- | --- |
| 7 | 100.0 | 0.0 | 0.000 |
| 9.5 | 100.0 | 4.2 | 0.042 |
| 11.5 | 100.0 | 8.3 | 0.083 |
| 12.5 | 100.0 | 20.8 | 0.208 |
| 13.5 | 100.0 | 25 | 0.250 |
| 14.5 | 98.2 | 25 | 0.232 |
| 15.5 | 98.2 | 29.2 | 0.274 |
| 16.5 | 94.5 | 37.5 | 0.320 |
| 17.5 | 89.1 | 45.8 | 0.349 |
| 18.5 | 87.3 | 54.2 | 0.415 |
| 19.5 | 87.3 | 58.3 | 0.456 |
| 20.5 | 81.8 | 62.5 | 0.443 |
| 22 | 76.4 | 62.5 | 0.389 |
| 24 | 69.1 | 62.5 | 0.316 |
| 25.5 | 63.6 | 66.7 | 0.303 |
| 26.5 | 63.6 | 75 | 0.386 |
| 27.5 | 61.8 | 75 | 0.368 |
| 28.5 | 56.4 | 83.3 | 0.397 |
| 29.5 | 56.4 | 91.7 | 0.481 |
| 30.5 | 50.9 | 91.7 | 0.426 |
| 31.5 | 49.1 | 91.7 | 0.408 |
| 32.5 | 43.6 | 95.8 | 0.394 |
| 33.5 | 41.8 | 95.8 | 0.376 |
| 34.5 | 32.7 | 95.8 | 0.285 |
| 35.5 | 32.7 | 100.0 | 0.327 |
| 36.5 | 29.1 | 100.0 | 0.291 |
| 37.5 | 27.3 | 100.0 | 0.273 |
| 39.0 | 25.5 | 100.0 | 0.255 |
| 40.5 | 23.6 | 100.0 | 0.236 |
| 43.5 | 21.8 | 100.0 | 0.218 |
| 46.5 | 18.2 | 100.0 | 0.182 |
| 47.5 | 16.4 | 100.0 | 0.164 |
| 48.5 | 10.9 | 100.0 | 0.109 |
| 50.5 | 9.1 | 100.0 | 0.091 |
| 54.5 | 7.3 | 100.0 | 0.073 |
| 58 | 5.5 | 100.0 | 0.055 |
| 59.5 | 3.6 | 100.0 | 0.036 |
| 63 | 1.8 | 100.0 | 0.018 |
| 67 | 0.0 | 100.0 | 0.000 |

## **Supplementary Figure 1: Enrolment procedure**

AF: atrial fibrillation, PSG: polysomnography
